# Supplementary material for: The value of diffusion tensor tractography delineating corticospinal tract in glioma in rat: validation via correlation histology
Source: PeerJ. 2019 Feb 13;7:e6453. doi: 10.7717/peerj.6453 (PMC6377590; doi:10.7717/peerj.6453)
Supplement: Supplemental Information 3 — Note: FA = fractional anisotropy; ADC = apparent diffusion coefficient; FDi = fiber density index; TA = tumoral areas; PA = peritumoral areas; Con = contralateral. [file peerj-07-6453-s003.docx]

| **Table S1.** FA, ADC and FDi values for tumoral and peritumoral areas and contralateral areas | | | |
| --- | --- | --- | --- |
| Parameter | FA | ADC | FDi |
| TA | 0.498±0.104 | 0.00125±0.00047s/mm^2^ | 0.947±0.498 |
| TA-Con | 0.698±0.061 | 0.00070±0.00010s/mm^2^ | / |
| PA | 0.589±0.072 | 0.00097±0.00021s/mm^2^ | 1.481±0.552 |
| PA-Con | 0.689±0.053 | 0.00072±0.00011s/mm^2^ | 5.325±1.077 |
| Note: FA=fractional anisotropy; ADC=apparent diffusion coefficient; FDi=fiber density index; TA=tumoral areas; PA=peritumoral areas; Con=contralateral. | | | |
