# Supplementary material for: The value of diffusion tensor tractography delineating corticospinal tract in glioma in rat: validation via correlation histology
Source: PeerJ. 2019 Feb 13;7:e6453. doi: 10.7717/peerj.6453 (PMC6377590; doi:10.7717/peerj.6453)
Supplement: Supplemental Information 4 — Note: rFA = ipsilateral FA/contralateral FA; rFDi = ipsilateral FDi/contralateral FDi; ADC ratio = ipsilateral ADC/contralateral ADC; Tumor cells(%) = percentage of glioma cells among the total number of cells in the peritumoral areas per high-powered field; Proliferation index(%) = fraction of proliferating cells in each peritumoral region; CST = corticospinal tract; Moderate (M), Severe (S). Three rats exhibited grade 1 injury, six rats demonstrated grade 2 injury, and eight rats displayed grade 3 injury. [file peerj-07-6453-s004.docx]

**Table S2**. Histological measures, CST injury grades and animal test scores in 17 rats and a summary of the MRI parameters in the peritumoral areas

| Number | rFA | rFDi | ADC ratio | Tumor cells (%) | | Proliferation index (%) | CST injury grade | Animal test (scores/grade) |
| --- | --- | --- | --- | --- | --- | --- | --- | --- |
| Animal 1 | 0.971 | 0.373 | 1.884 | 0.39 | 0.14 | | Grade1 | 6/M |
| Animal 2 | 0.911 | 0.3 | 1.46 | 0.36 | 0.25 | | Grade2 | 4/ M |
| Animal 3 | 0.924 | 0.27 | 1.625 | 0.48 | 0.22 | | Grade2 | 3/ M |
| Animal 4 | 0.971 | 0.322 | 1.119 | 0.39 | 0.15 | | Grade1 | 5/ M |
| Animal 5 | 0.894 | 0.256 | 1.268 | 0.52 | 0.21 | | Grade2 | 3/ M |
| Animal 6 | 0.803 | 0.234 | 1.129 | 0.54 | 0.32 | | Grade3 | 1/S |
| Animal 7 | 0.708 | 0.361 | 1.25 | 0.57 | 0.35 | | Grade3 | 2/ S |
| Animal 8 | 0.731 | 0.324 | 1.379 | 0.53 | 0.47 | | Grade3 | 2/ S |
| Animal 9 | 0.909 | 0.228 | 1.481 | 0.51 | 0.25 | | Grade2 | 4/ M |
| Animal 10 | 0.884 | 0.259 | 1.236 | 0.50 | 0.22 | | Grade2 | 3/ M |
| Animal 11 | 0.864 | 0.224 | 1.269 | 0.55 | 0.47 | | Grade3 | 3/ M |
| Animal 12 | 0.778 | 0.23 | 1.305 | 0.56 | 0.49 | | Grade3 | 1/ S |
| Animal 13 | 0.821 | 0.18 | 1.26 | 0.62 | 0.59 | | Grade3 | 1/ S |
| Animal 14 | 0.957 | 0.331 | 1.226 | 0.40 | 0.19 | | Grade1 | 5/ M |
| Animal 15 | 0.859 | 0.263 | 1.587 | 0.48 | 0.27 | | Grade2 | 3/ M |
| Animal 16 | 0.848 | 0.279 | 1.352 | 0.56 | 0.29 | | Grade3 | 1/ S |
| Animal 17 | 0.719 | 0.164 | 1.211 | 0.61 | 0.55 | | Grade3 | 1/ S |

Note: rFA= ipsilateral FA/contralateral FA; rFDi= ipsilateral FDi/contralateral FDi; ADC ratio= ipsilateral ADC/contralateral ADC; Tumor cells(%)= percentage of glioma cells among the total number of cells in the peritumoral areas per high-powered field; Proliferation index(%)= fraction of proliferating cells in each peritumoral region; CST= corticospinal tract; Moderate (M), Severe (S). Three rats exhibited grade 1 injury, six rats demonstrated grade 2 injury, and eight rats displayed grade 3 injury.
