# Supplementary material for: The value of diffusion tensor tractography delineating corticospinal tract in glioma in rat: validation via correlation histology
Source: PeerJ. 2019 Feb 13;7:e6453. doi: 10.7717/peerj.6453 (PMC6377590; doi:10.7717/peerj.6453)
Supplement: Supplemental Information 5 — Note: FA = fractional anisotropy; ADC = apparent diffusion coefficient; FDi = fiber density index; TA = tumoral areas; PA = peritumoral areas; Con = contralateral;. [file peerj-07-6453-s005.docx]

| **Table S3.** Comparison of the FA, ADC and FDi values among tumoral and peritumoral areas and contralateral areas | | | |
| --- | --- | --- | --- |
|  | FA | ADC | FDi |
|  | P value | P value | P value |
| TA and TA-Con | 0.0023 | 0.0158 | <0.0001 |
| PA and PA-Con | 0.0105 | 0.0004 | <0.0001 |
| TA and PA | 0.0002 | 0.0019 | <0.0001 |
| Note: FA=fractional anisotropy; ADC=apparent diffusion coefficient; FDi=fiber density index; TA=tumoral areas; PA=peritumoral areas; Con=contralateral; | | | |
