# Supplementary figures and images for: The value of diffusion tensor tractography delineating corticospinal tract in glioma in rat: validation via correlation histology
Source: PeerJ. 2019 Feb 13;7:e6453. doi: 10.7717/peerj.6453 (PMC6377590; doi:10.7717/peerj.6453)

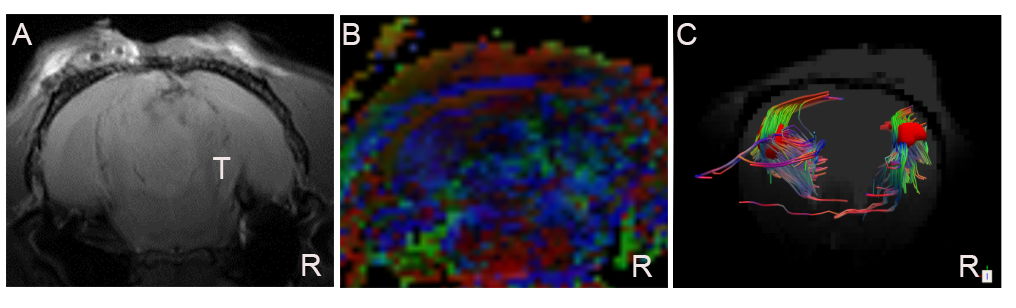

Supplement: Supplemental Information 6 — A, T2W image showing a tumor (T indicates the tumor). B, Representative FA color imaging. The anisotropy signal displayed a slight decrease relative to the corresponding tracts in the left hemisphere. C, Representative DTT imaging, top view. The right CST is slightly displaced by the lesion; R = right side. [file peerj-07-6453-s006.png]
